# Unveiling the Biotechnological and Agronomic Potential of Amazonian Fruit Species from the Genus *Eugenia* (Myrtaceae): Functional Traits and Applied Perspectives

**DOI:** 10.3390/plants15040646

**Published:** 2026-02-19

**Authors:** Pedro Paulo dos Santos, Josiane Celerino de Carvalho, Elmer Viana Gonçalves, Karen Cristina Pires da Costa, Fernanda Adrielle da Silva Rocha, Acácio de Andrade Pacheco, Hector Henrique Ferreira Koolen, Jaime Paiva Lopes Aguiar, Andreia Varmes Fernandes, José Francisco de Carvalho Gonçalves

**Affiliations:** 1 Laboratory of Plant Physiology and Biochemistry, National Institute of Amazonian Research—(INPA), Manaus 69060-020, AM, Brazil; santospp@gmail.com (P.P.d.S.); elmergoncalves@outlook.com (E.V.G.); varmes@inpa.gov.br (A.V.F.); 2 Bionorte Postgraduate Program, University of Amazonas State, Manaus 69065-020, AM, Brazil; hkoolen@uea.edu.br; 3 Faculty of Agronomy, Institute of Studies in Agrarian and Regional Development—(IEDAR), Federal University of South and Southeast of Pará—(UNIFESSPA), Maraba 68500-000, PA, Brazil; karencosta@unifesspa.edu.br; 4 Metabolomics and Mass Spectrometry Research Group, University of Amazonas State, Manaus 69065-020, AM, Brazil; fasr.mbt23@uea.edu.br; 5 Federal Institute of Education, Science and Technology of Pará—(IFPA), Maraba 68502-120, PA, Brazil; acacio.pacheco@ifpa.edu.br; 6 Laboratory of Physical Chemistry of Food National Institute for Amazonian Research—(INPA), Manaus 69060-020, AM, Brazil; jaguiar@inpa.gov.br

**Keywords:** domestication, morphofunctional traits, chemical composition, planting systems, food

## Abstract

*Eugenia* (Myrtaceae) is a highly diverse genus of fruit trees native to the Amazon with remarkable potential for food, nutritional, and biotechnological applications. This review synthesizes the current knowledge on morphofunctional traits, ecological strategies, and genetic resources that make several *Eugenia* species promising candidates for domestication and cultivation. Its main attributes include shrubby growth habits, racemose inflorescences, nutrient-rich fruits with few seeds, recalcitrant yet viable propagules, and wide distribution across the Americas. Their molecular and phytochemical diversity suggests applications in food systems, pharmaceuticals, and bioindustries. However, key challenges persist, such as irregular fruiting, postharvest perishability, limited germplasm conservation in degraded areas, prospecting biotechnological applications such as antioxidants, and insufficient genomic characterization. By addressing these gaps, *Eugenia* domestication could contribute to food security, sustainable agriculture, and the bioeconomy of remote Amazonian regions, thereby positioning this genus as a strategic resource in the face of biodiversity loss.

## 1. Introduction

The contrast between the vast diversity of Amazonian fruit trees and the high prevalence of malnutrition affecting approximately five million people in 2021 [1] highlights the urgency of strengthening food security and sovereignty through scientific knowledge accessible to local communities. For over 11 millennia, native plants have been central to traditional medicinal, nutritional, and cultural practices [2], yet many are now facing extinction because of rapid climate change, often without scientific documentation [3]. Therefore, advancing research on cultivation, germination, genetic resources, and biochemical potential is essential for the development of novel plant-based bioproducts [4]. This demand converges with a global trend toward greater consumption of plant-based foods, particularly fruits and vegetables, owing to their health-promoting benefits beyond nutrition [5].

The Myrtaceae family, which stands out in Brazil as a biodiverse group of angiosperms and is the third largest in endemism, produces fleshy fruits such as Surinam cherry and guava [6]. In the Brazilian Amazon, there are approximately 265 representatives organized into 13 genera; however, few studies systematized them in terms of botany, technology, use, and agronomy [7]. The genus *Eugenia* owns approximately 1100 species identified in the Neotropics and approximately 380 species in Brazil, of which 300 are endemic [8,9]. In addition, a considerable number of representatives have intraspecific variations that allow for domestication and prospecting for new foods, and cosmetics to combat ailments such as obesity [10].

Studies on the possibility of cultivating and generating bioproducts based on the fruits and vegetative organs of species of *Eugenia* have been conducted in recent decades, contributing to the bioprospecting of some fruit species [11]. For the establishment of crops of any species and management itself, it is important to have information accessible to farmers regarding the mobilization of primary reserves during germination [12], which is a challenge for the knowledge of the physiology of poorly bioprospected representatives of the genus *Eugenia* occurring in the Brazilian Amazon. In addition to the production of fruit for food and food supplements [6,13], the essential oils and extracts present in the leaves and twigs are among the potential species from the group that have already been studied in the Amazon and which justify cultivation [14,15] and the relationship with biological activities manifested in the form of anti-inflammatory, antimicrobial, antioxidant, cytotoxic, anticancer [16,17] and hypoglycemic activities [18].

Still focusing on essential oils, Costa et al. (2020) [16] and Jeronimo et al. (2021) [15] point out that the species studied in the Brazilian Amazon until those years were *Eugenia biflora* (L.) DC. (1828), *Eugenia egensis* DC. (1828), *Eugenia flavescens* DC. (1828), *Eugenia patrisii* Vahl. (1798), *Eugenia polystachya* Rich. (1792), *Eugenia protenta* McVaugh (1969), *Eugenia punicifolia* (Kunth) DC. (1828) and *Eugenia stipitata* McVaugh (1956), comprising eight species with the potential for generating crops and biotechnology, which are addressed in this review.

Considering that the genus *Eugenia* can be used in intercropped and agroforestry systems, especially under forestry restoration conditions, and that the implementation of this system can offer an alternative means of circumventing Amazonian food insecurity, it is important to understand the biological characteristics of these species and their current state of research. Considering that the genus *Eugenia* can be used in intercropped and agroforestry systems, particularly under forest restoration conditions, and that these systems may offer alternative pathways to mitigate Amazonian food insecurity, a clear understanding of the biological characteristics and current research status of this species is essential. Thus, a comprehensive and integrative synthesis that bridges ecological distribution, morphofunctional and physiological traits, and documented biotechnological and agronomic applications of Amazonian *Eugenia* species remains lacking. Addressing this gap is particularly relevant given their potential contributions to food security, income generation, and ecosystem recovery. In this review, we aimed to provide an integrative and application-oriented synthesis of current scientific knowledge on *Eugenia* species (Myrtaceae) occurring in the Brazilian Amazon. Specifically, we compiled and critically analyzed information on species distribution and ecology, domestication status, morphofunctional traits, genetic resources, chemical composition, and documented biotechnological and agronomic applications. By adopting this comparative and integrative framework, this review identifies key knowledge gaps and highlights the functional attributes that are directly relevant to crop development, sustainable production systems, and forest restoration strategies in the Amazon.

## 2. Domestication and Ecological Traits

*Eugenia stipitata* has at least two subspecies related to geographical isolation by rivers in the western Amazon: *E. stipitata* subsp. *stipitata* and *E. stipitata* subsp. *sororia*. However, the fruits of the latter subspecies showed a particular pattern among the representatives of the *Eugenia* genus: a high number of seeds (6–15), and a mesocarp diameter of 8–10 cm [19]. Consequently, this subspecies is the type cultivated in home gardens, which has shown potential for forestry cultivation in recent times [20], suggesting that the possible origin of this subspecies is domestication.

**Figure 1:**
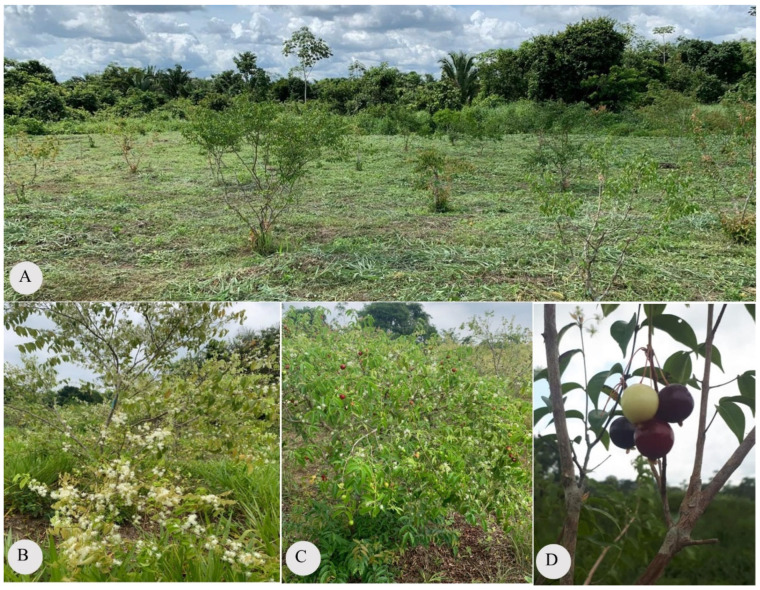
Illustrative photographs of *Eugenia patrisii* cultivation in the city of Marabá (Pará-Brazil), in the year 2024. (**A**) General view of the arrangement of individuals; (**B**) Flowering plant during the harvest period; (**C**) Individuals with fruit at different stages of ripeness; (**D**) Fruit at different stages of ripeness.

**Figure 2:**
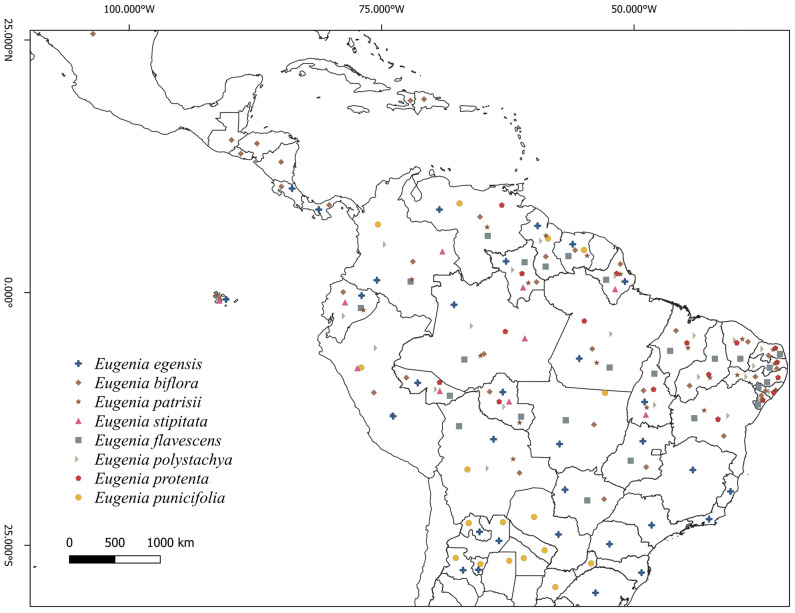
Map of the geographical distribution of *Eugenia* species on the American continent. *Eugenia biflora*, *E. egensis*, *E. flavescens*, *E. patrisii*, *E. polystachya*, *E. protenta*, *E. punicifolia* and *E. stipitata.* Based on Govaerts (2023) [9].

*Eugenia patrisii*, under cultivation conditions, also demonstrates the existence of intraspecific variations called phenotypes [11] (Figure 1). The main distinctions pointed out by the authors were also related to biomass accumulation, quantity of propagule production, and maturity of fruit production (Figure 1B–D). For *E. biflora*, *E. egensis*, *E. flavescens*, *E. polystachya*, and *E. protenta*, no studies have suggested variations in propagation structures or domestic selection. In terms of geographical distribution, the species is exclusively American and scattered throughout North, Central, and South America (Figure 2). The species *Eugenia flavescens*, *E. patrisii*, *E. polystachya*, *E. protenta* and *E. stipitata* occur exclusively in the Amazon ecosystem [9]. *E*. *biflora* and *E. egensis* are the most widely distributed in the Americas from north to south. *E. punicifolia* is found in the Amazon, Caatinga, Cerrado, and Atlantic rainforests [8].

### Ecological Traits

Exposure to high solar radiation inhibits the growth and development of the vegetative body and phenology of *Eugenia stipita* and *E. punicifolia* when cultivated [21]. A solution for maintaining crops of these species is to plant them in a shading system, wood, or forest, which reduces the incidence of light on the specimens.

The leaves’ extracts were tested and used as allelopathic biological agents for the germination and initial growth of *Lactuca sativa* L. and *Solanum lycopersicum* L. seedlings [14]. The chemical similarity between the compounds in the species of the genus was proven; however, the variation in concentrations allowed the establishment of different levels of damage to the infected species. The fruits of *E. stipitata* are the main hosts for fly species and are also used for mating and oviposition [22].

Pollination syndromes in *Eugenia* are predominantly caused by bees belonging to the Apidae family [23]. *E. stipitata* has been recorded in *Apis mellifera*, *Eulaema mocsaryi*, *E. bombiformis*, *Melipona lateralis*, *Megalopta* sp., and *Melipona pseudocentris* [24]. The other clades discussed did not include studies on gamete exchange, which is extremely important for characterizing their potential for establishing crops. Therefore, the fruit dispersal syndrome of *Eugenia punicifolia* may be caused by more than one species of terrestrial birds from the Tinamidae family [23]. In *E. egensis* and *E. flavescens*, the distance from the mother plant is mediated by monkeys from different groups, and *E. patrisii*, dispersal is mediated by birds and rodents; however, the specific species involved remain unclear [25]. However, according to these authors, there is a clear gap in information regarding the animals that carry out the dispersal syndrome of *Eugenia* fruits in northern South America.

In *Eugenia punicifolia*, the insect species identified as pollinators were *Apis mellifera*, *Melipona quadrifasciata anthidioides*, *Trigona spinipes*, and *Partamona* sp., although the species can also self-pollinate with a lower frequency of fruit formation, compromising the breeding system [26]. Apparently, in representatives of the Myrtaceae family, xenogamy is the most efficient reproductive strategy for fruit generation, thus interactions with insects stand out from others as a pollination syndrome [27].

Recently, studies have indicated a striking characteristic that contradicts the apparent morphological homogeneity of *Eugenia*, floral heterochrony, which consists of subtly different stages in the ontogeny and evolution of species and sections [26]. In this study, we analyzed samples of *Eugenia flavescens*, *E. punicifolia* and *E. stipitata* and 17 other species, pointing out subtle particularities.

The domestication potential of *Eugenia* species lies in the interplay between their ecological traits and human selection pressures. Variations in fruiting cycles, pollination syndromes, and dispersal strategies not only shape species’ adaptation but also define their suitability for cultivation under agroforestry systems. Recognizing these ecological patterns in parallel with domestication history can guide targeted breeding strategies, particularly for identifying species with greater resilience to environmental fluctuations and higher potential for consistent yields. Together, these ecological and domestication-related aspects provide an environmental and management context in which the morphofunctional traits of Amazonian *Eugenia* species are expressed and interpreted, serving as a foundation for the following section.

## 3. Morphofunctional Traits

Building upon the ecological and domestication context outlined above, this section focuses on the morphofunctional traits of *Eugenia* species, emphasizing structural and physiological attributes directly related to reproduction, germination, and plant performance.

### 3.1. Botany

**Figure 3:**
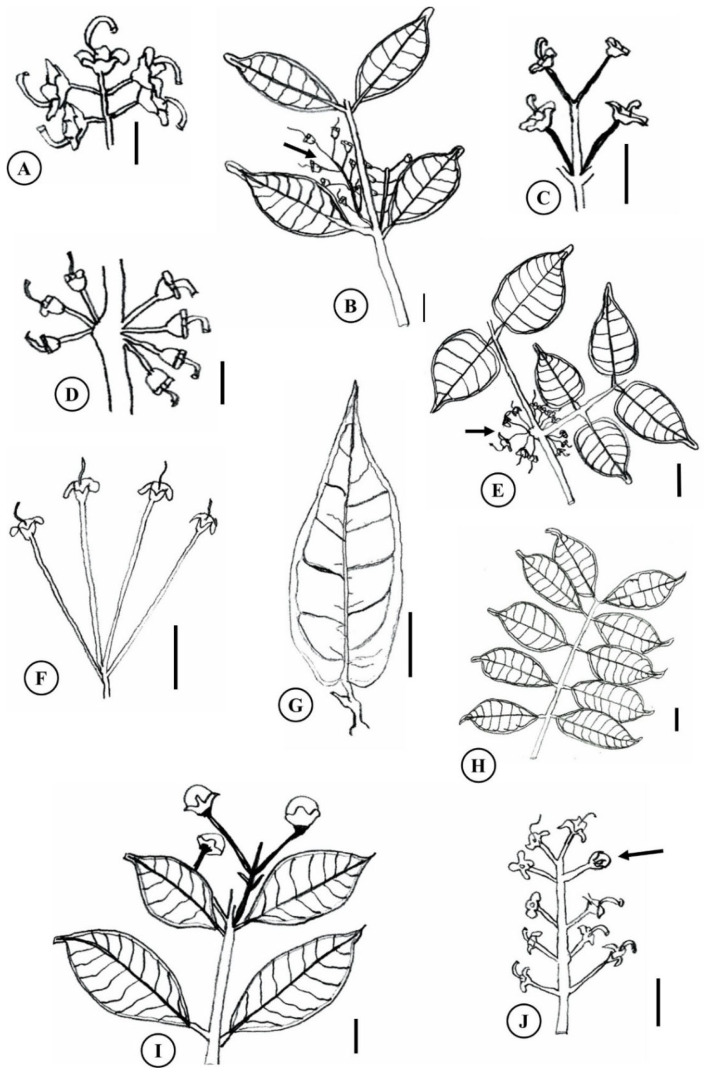
Morphology of Amazonian *Eugenia*. (**A**) inflorescence with fertilized flowers of *E. biflora*; (**B**) leaf branch of *E. egensis*, with opposite axillary inflorescence (arrow); (**C**) inflorescence of *E. punicifolia* with fertilized flowers; (**D**) inflorescence with fertilized flowers of *E. flavescens*; (**E**) leaf branch of *E. protenta* with axillary fertilized flowers (arrow); (**F**,**G**) inflorescence with fertilized flowers and leaf of *E. patrisii*; (**H**) *E. stipitata* leaf branch; (**I**,**J**) *E. polystachya* leaf branch with inflorescence in axillary flower buds and inflorescence with fertilized flowers and flower buds (arrow). Scale 1 cm. Based on Govaerts (2023) [9].

Although morphological standardization of some *Eugenia* sections remains unclear, key clades include *Pseudeugenia* Mazine & Faria, *Racemosa* O. Berg, and *Umbellatae* sensu, according to Mazine et al. (2014) [27]. The first is characterized by flowers with filiform, deciduous bracteoles and large, edible fruits, such as *E. patrisii* and *E. stipitata* [28]. The second features flowers in raceme or panicle inflorescences, with axes coinciding with elongated pedicels, as observed in *E. biflora* (Figure 3A) and *E. polystachya* [29]. Finally, *Umbellatae* encompasses single flowers or those in racemes, fascicles, or glomerules, with pedicels wider than the floral whorls, such as *E. egensis*, *E. flavescens*, *E. protenta*, and *E. punicifolia* (Figure 3C,D) [28]. According to Mazine et al. (2014) [27], the inflorescence structure in *Eugenia* varies subtly within and between species, posing challenges to clade delimitation.

Herbarium records of properly identified botanical samples from the Amazon and Brazil reveal varying numbers of specimens, reflecting distribution patterns and collection efforts over the past two centuries: *E. biflora* (449), *E. egensis* (400), *E. flavescens* (394), *E. patrisii* (376), *E. polystachya* (75), *E. protenta* (40), *E. punicifolia* (4375), and *E. stipitata* (39) [8]. *E. stipitata* is one of the best-known species among Amazonian populations, yet it has the fewest records [20]. No germplasm banks for these species exist in the Amazon or South America, underscoring challenges in accessing genetic material.

### 3.2. Morphoanatomy

*Eugenia* species occurring in the Brazilian Amazon exhibit habits ranging from shrubby to arboreal, depending on environmental conditions such as light availability or competition [30,31]. In non-forest vegetation, *E. biflora*, *E. flavescens*, *E. patrisii*, *E. polystachya*, *E. punicifolia*, and *E. stipitata* typically grow as shrubs, whereas *E. egensis* and *E. protenta* are trees [32].

Trunks feature a rhytidome that peels off in juxtaposed plates of varying textures, releasing easily detachable bran upon touch. Leaves are simple, opposite (Figure 3B,E,G,H), papery to fibrous in texture, with prominent brochidodromous venation on both surfaces. Leaf blades vary from oval to elliptical–lanceolate and host conspicuous translucent glands (Figure 3B,E,G,H) [32].

**Figure 4:**
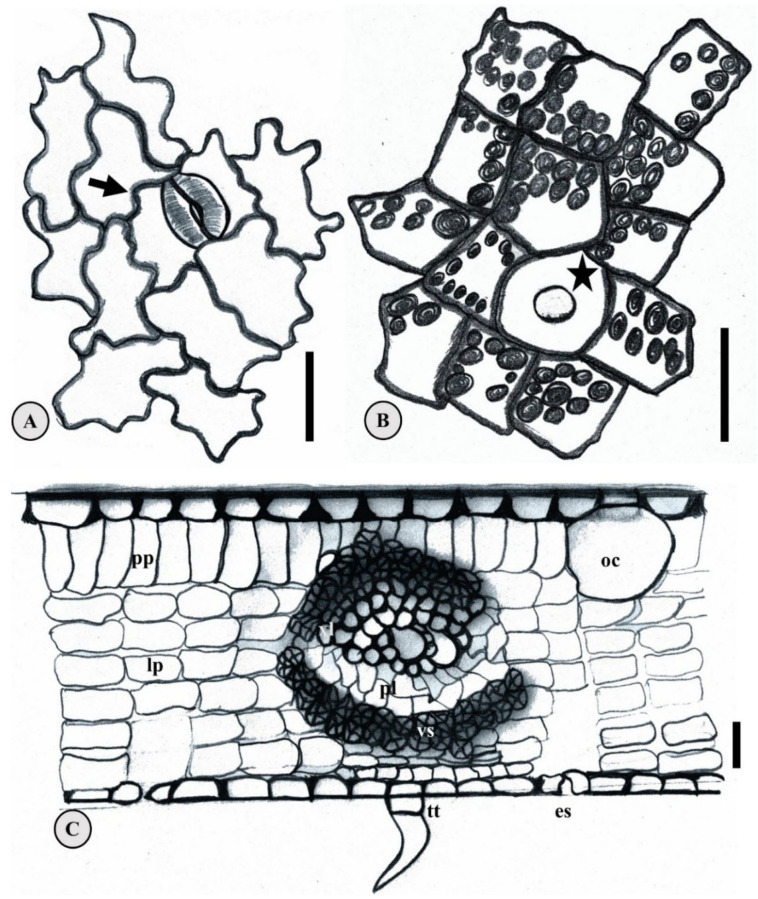
Anatomical structures of *Eugenia patrisii.* (**A**) Abaxial leaf epidermis, showing a paracytic stomatal complex (arrow); (**B**) amyliferous parenchyma of the seed cotyledon indicating an oil cell (star); (**C**) Transection cross of the leaf mesophyll showing collateral vascular bundler. es. Estomata; lp. Lacunar parenchyma; oc. Oil cavity; pl. phloem; pp. palisade parenchyma; tt. tector trichomes vs. vascular sheath. Scale 10 µm. Based on Alvarez and Silva (2012) [33].

Leaf anatomy may elucidate the phylogenetic origins of four species, including *E. biflora*, *E. flavescens*, and *E. patrisii* [33]. Key characters include sinuosity in the parietal walls of epidermal cells on both adaxial and abaxial surfaces (Figure 4A), thick leaf cuticle, unicellular tector trichomes (Figure 4C), hypostomatic leaves (Figure 4A,C), paracytic (Figure 4A) and anomocytic stomata, lipid-containing secretory cavities (Figure 4C), idioblasts secreting phenolic compounds, and collateral vascular bundles (Figure 4C). Jorge et al. (2000) [34] analyzed leaf morphology in three Amazonian Myrtaceae species, including *E. punicifolia*. The main traits highlighted were obovate-to-elliptical leaf blade shape, undulate parietal walls of epidermal cells, one to two layers of palisade parenchyma, oil cavities, striated cuticle, bicollateral central vascular bundle, hypostomatic leaves, and anomocytic stomata.

Morphoanatomy of the mature *E. stipitata* seed was described to elucidate species of germination [35]. The propagule is starchy and pachychalazal, featuring an undifferentiated eugenoid embryo axis. The seed coat comprises three tissue types: reserve parenchyma with starch and oil glands on the cotyledons (Figure 4B), juxtaposed exotesta, mesotesta with loose sclerenchyma and vascular bundles, and endotesta with lax sclerenchymatous cells. Anatomical descriptions of *E. egensis*, *E. polystachya*, and *E. protenta* seeds remain unavailable, highlighting a research gap.

### 3.3. Flowers

Flowers of Amazonian *Eugenia* species occur singly or in axillary/terminal inflorescences of raceme or fascicle types (Figure 1) [30]. They bear deciduous bracts and bracteoles that may persist in post-anthesis [9]. Flowers are pedunculate with open calyces, four sepals, four petals, and bi- or tri-locular ovaries [8] (Figure 3). They are actinomorphic, dichlamydial, and dialipetalous (Figure 3), with numerous stamens in the androecium. Inflorescence details are summarized in Table 1.

### 3.4. Fruits

**Figure 5:**
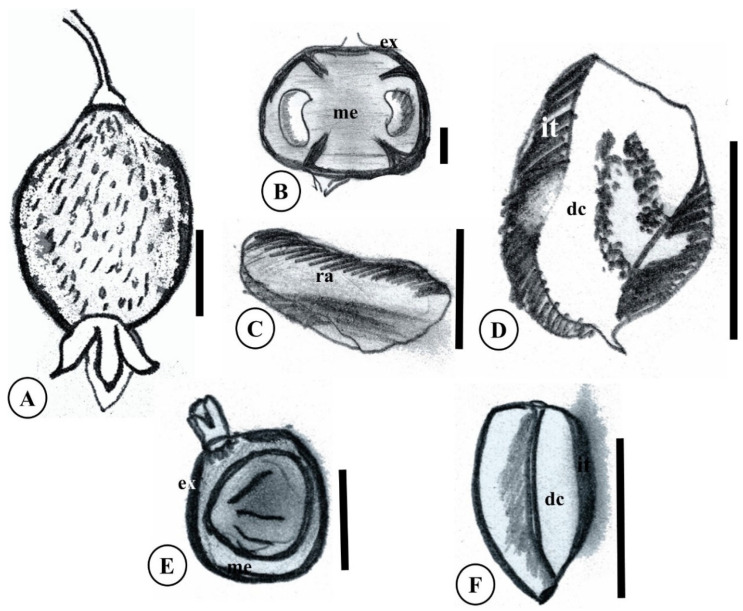
Fruits and seeds of *Eugenia* amazonica. (**A**) Fruit of *E*. *biflora* showing glands on the exocarp; (**B**) Fruit of *E. stipitata* showing seeds; (**C,D**) seed of *E. stipitata* in external and internal morphology; (**E**) Fruit of *E. patrisii*, showing a single seed; (**F**) Seed of *E. patrisii*, in section. dc. Dense cotyledon; ex. Exocarp; it. Integument; me. Mesocarp; ra. Raphe. Scale 1 cm. Based on Anjos and Ferraz (1999) [41] and Souza et al. (1999) [32].

Fruits of *Eugenia* species occurring in the Brazilian Amazon are bacaceous, varying in morphology, size, color, consistency, and dehiscence, often with a crown of persistent calyx lobes (Figure 1D and Figure 5A) [30]. Exocarps of these species are typically dark-colored, likely due to high anthocyanin, flavonoid, and polyphenol contents [38]. The mesocarp is generally juicy and acidic, with a rancid, aromatic odor characteristic of Myrtaceae (myrtle scent) [13] (Figure 5B). Acidity gradients exist among the reviewed species, with *E. stipitata* being the least palatable for fresh consumption. Despite the absence of anatomical or developmental studies on Amazonian *Eugenia* fruits, secretory glands typical of Myrtaceae leaves (e.g., in *E. biflora*; Figure 5A), which produce terpenes including volatile essential oils, are likely responsible for fruit aroma [39]. For cultivation aimed at fruit production, all species may be climacteric, showing limited post-harvest modifications in palatability; fruits require rapid consumption, drying, or refrigeration after detachment from the parent plant, as observed in *E. stipitata* [40].

Regarding consumption, the exocarp and mesocarp constitute the edible pulp, while the endocarp adheres to the seed, as observed in *E. patrisii* (Figure 5E) and described by Araújo et al. (2021) [13] for *E. stipitata* (Figure 5B). Pulp taste is sour, ranging from bitter to sweet, with a distinctive myrtle aroma even when ripe [6,42]. Fruit characteristics are summarized in Table 2. Fruits listed in Table 2 are typically globose and, except for *E. stipitata*, contain no more than five seeds.

Regarding biometrics, Ferreira (1992) [43] characterized total weight, peel and pulp weight, average fruit diameter and length, seed number, and seed weight for *E. stipitata*, noting value oscillations. For *E. patrisii*, Pacheco et al. (2021) [11] quantified fruits produced per individual and average fruit mass under cultivation. Physicochemical and calorimetric profiling of *E. stipitata* fruits revealed infraspecific variation [44]. According to these authors, fruit dimensions, weights, soluble solids, pH, peel and flesh color, chromaticity, and luminosity diverged significantly among 33 samples from distinct geographical origins in the Brazilian Amazon.

### 3.5. Seeds

Myrtaceae seeds are distinctive and inform tribal and generic classifications, including *Eugenia*, which feature dense, conferruminate cotyledons without a distinct embryonic axis (Figure 5D,F) [19]. Morphological studies of Amazonian species have focused on *E. stipitata*, with its monoembryonic, recalcitrant, pseudomonocotyledonous seeds (Figure 5D) [35,41,45]. Seeds exhibit distinct integument, micropyle, raphe, and hilum, characteristic of the campylotropous type with slightly curved embryos (Figure 5D) [35,46]. Integument of coloration varies among individuals of the same species, but shapes are consistent, as in *E. patrisii* and *E. stipitata* (Figure 5D,F).

**Figure 6:**
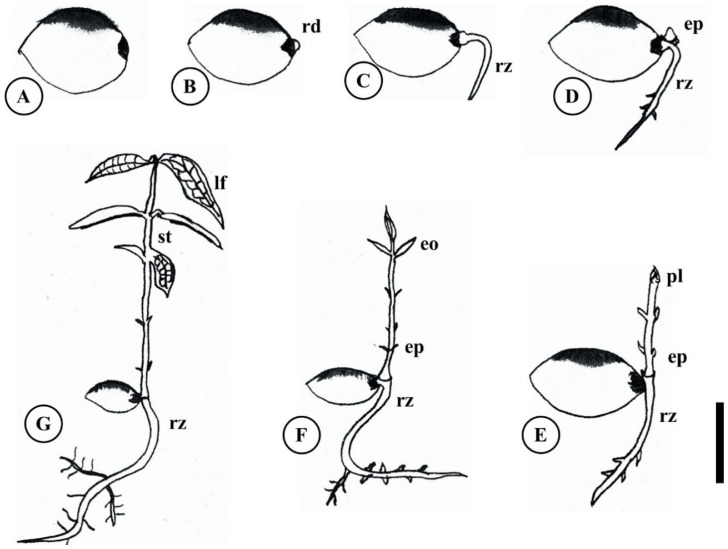
Stages of germination in *Eugenia patrisii.* (**A**) meristematic growth of the hypocotyl–root axis; (**B**) protrusion of the radicle; (**C**) growth of the primary root; (**D**) emergence of the epicotyl; (**E**) elongation of the root and epicotyl, and differentiation of the plumule and cataphylls; (**F**) distension of the opposite eophylls; (**G**) formation of the seedling. eo. Eophylls; ep. Epicotyl; lf. Leaf; pl. Plumule; rd. Radicle; rz. Primary root; st. Stem. Scale 1 cm. Based on Santos et al. (2025) [54].

Seed anatomy of *E. egensis*, *E. flavescens*, and *E. punicifolia* reveals unitegmic coats in the first two—pachychalazal, exalbuminous, with single eugenoid embryos and thick, fleshy ripe cotyledons [46]. The authors used morphology, cell wall traits, and exomesotestal fibers to inform phylogeny. A single, vibrio-shaped eugenoid embryo is common in Brazilian *Eugenia* species, with separate or fused (conferruminate) cotyledons and a slightly protruding or included hypocotyl–radicle axis (Figure 5D,F) [47]. In *E. stipitata*, cotyledons are fused, with an indistinct hypocotyl–radicle axis evident only as a subtle prominence [35]; fused cotyledons also occur in *E. protenta* [8]. *E. patrisii* embryos share this composition (Figure 6).

Nuc Ludghadha and Proença (1996) [23] identified gaps in *Eugenia* embryonic development knowledge, partly due to recalcitrant seeds’ resistance to anatomical processing, hindering ontogenetic visualization. Selective pressures on eugenoid embryos favor nutrient reserves for viable seedlings. In these exalbuminous *Eugenia* seeds, cotyledons serve as the primary storage site, rich in amyliferous tissue (Figure 5D,F) [35,46]. Schizogenous secretory cavities containing essential oils may also occur, as observed in *E. patrisii* cotyledons [23] (Figure 4B).

*E. stipitata* seeds are desiccation-sensitive yet resistant to mechanical damage, enabling meristematic tissue regeneration [41]. This cellular totipotency likely relates to hormonal regulation, maturity, humidity, and temperature [48,49]. Amorim et al. (2020) [50] position *E. stipitata* as a model for neotropical Myrtaceae seed physiology. Evolutionarily, its totipotency may link to polyembryony (a group apomorphy) [48], high seed counts per fruit in other *Eugenia* species, and/or dispersal strategies [23].

### 3.6. Germination and Propagation

*Eugenia* species exhibit seed peculiarities that directly influence germination and seedling/sapling viability, including, as detailed previously, tissue regeneration and embryonic totipotency from mass reduction or fragmentation, hypogeal cryptocotyledonous germination [49,50], pre-maximum fruit maturity for germination readiness [51], dissection tolerance [52] and germination at temperatures up to 35 °C [53]. Under controlled conditions, *E. stipitata* achieved a germination percentage (TG) of 62% ± 16.4; fragmentation into two poles increased this to 94% ± 4.18 via regeneration. Mean germination time (MGT) was 119.6 ± 14.13 days, and germination speed index (GSI) was 0.008 ± 0.0011 seeds day^−1^ [48]. For this species, seed dimensions (length × width × thickness) averaged 1.06 × 0.88 × 0.62 cm, with fresh and dry weights of 0.49 g and 0.17 g, respectively [41]. Water content was 62%, with germination initiating ~50 days post-sowing. *E. patrisii* showed MGT of 13.4 ± 1.8 to 17.8 ± 0.3 days, germination percentages of 12.5 ± 7.7 to 100 ± 0.0%, MVG of 0.1 ± 0.0, and dimensions (length × width × thickness) ranging from 0.28 ± 0.3/0.41 ± 1.8/0.29 ± 0.6 to 0.9 ± 0.7/1.4 ± 1.1/0.95 ± 1.0 cm [54].

Germination stages in *E. stipitata* comprised six phases: (1) meristematic growth of the hypocotyl–root axis; (2) radicle protrusion; (3) primary root growth; (4) epicotyl emergence; (5) root and epicotyl elongation; and (6) seedling formation [41]. In *E. patrisii*, the final two phases showed distinctions, subdividing into (5.2) plumule and cataphyll differentiation; and (6.1) opposite eophyll distension (Figure 6) [54]. Studies over the past two decades on genus representatives indicate sustained germination percentages under adverse conditions, such as those from tropical climate change (drought, high humidity, temperature fluctuations, and reduced substrate diversity) [50].

*E. stipitata* exhibits desiccation intolerance but enters dormancy under water stress [48]. Mendes and Mendonça (2012) [55] characterized pre-germination treatments, influencing viable seedling production, including leaching, partial integument removal, and fractionation. Seeds remained viable up to 50 days submerged; integument suppression reduced germination time from 91 to 48 days; seed division neither prevented nor altered germination rates. Germination data for *E. biflora*, *E. egensis*, *E. flavescens*, *E. polystachya*, *E. protenta*, and *E. punicifolia* remain unavailable, necessitating further studies.

Morphological and anatomical traits, such as secretory cavities, seed recalcitrance, and regenerative totipotency, extend beyond diagnostics; they directly impact propagation of success and postharvest handling. Integrated with biochemical and physiological data, these features reveal adaptive strategies exploitable for domestication. For example, *E. stipitata* seed totipotency, coupled with rich reserve metabolism, offers dual potential for genetic conservation and bioactive compound extraction, underscoring the need to align morphofunctional insights with crop development objectives.

## 4. Genetic Resources

Some Amazonian Myrtaceae occur in vivo gene banks within traditional crops, such as *Myrciaria dúbia* [56] and *Eugenia stipitata* [44]. However, in vitro, germplasm banks are lacking. These could facilitate genetic characterization, infraspecific variation detection, and propagation of crop-adapted genotypes. Considerable genotypic information for *Eugenia* species derives from fruit physicochemical profiles, as established for *E. stipitata* and *E. patrisii* [11,44]. Nonetheless, no studies document nuclear or plastidial DNA polymorphism, hindering genetic improvement patterns. Few characterizations exist for Brazilian Myrtaceae nuclear and plastid genomes, which could elucidate inter- and intraspecific divergences at molecular (chemical polymorphism) or phenotypic levels (e.g., coloration), culminating in extract and essential oil bioactivities [57,58].

Wang and Ding (2023) [59], showed that *Eucalyptus* phenology, a Myrtaceae genus with annual cycles, is regulated by nuclear genes such as AtFT (*Arabidopsis* T floral locus stimulator), PtFT1 (*Populus* T floral locus stimulator), and ELFY (floral meristem regulator), manipulable to advance/delay flowering, fruiting and growth. No equivalent studies exist for *Eugenia*, though similar gene roles in life-cycle phenology are plausible.

For genetic improvement of fruiting Amazonian *Eugenia*, targeting seasonal/continuous yields and rational cropping—molecular data are scarce, relying on chloroplast DNA for interspecific divergence [60]. Given their wide Amazonian distribution, genetic, edaphic, and microclimatic variations likely influence nutrition, fruit production, and chemical composition.

*Eugenia* genetic resources remain underexplored, with limited molecular data impeding superior genotype identification. Linking intraspecific variation to fruit quality, oil composition, or ecological performance could yield selection markers. Integrating genomics, metabolomics, and phenomics would support holistic approaches for situ conservation and targeted breeding. Advancing molecular characterization is thus essential to convert biodiversity into agronomic value.

Myrtaceae have advanced omics understanding in plants. *Syzygium* and *Melaleuca* leaf essential oils feature 25 metabolites (e.g., butanal, cyclopentanol, nonanal, octanal) as species markers, dominated by ketones and aryl-aldehydes, potentially applicable to *Eugenia* [61]. In *Syzygium*, eugenol biosynthesis genomics revealed 116 phenylpropanoid genes (PAL, C4H, 4CL, HCT, C3H, CSE, COMT, CCoAOMT, F5H, CCR, CAD) across seven chromosomes [62].

Metabolomically, Myrtaceae intraspecific varieties differ in primary/secondary constituents, such as seed reserve mobilization during germination or leaf essential oils [63,64]. In *E. patrisii* seeds, starch and soluble carbohydrates (glucose, fructose, sucrose) degrade rapidly from germination onset in phenotype Ph6, but slowly initially then accelerating in Ph2/Ph3 [64]. In *Psidium guajava*, leaf volatile oils vary in β-caryophyllene (Caxcana: 16.46%; S-56: 23.6%); in silico/molecular docking links these to interactions with CB2, PPARα, BAX, BCL2, and AKT1 proteins, relevant to inflammation in neurodegenerative diseases [63].

Eugenol and β-caryophyllene, bioactive volatiles in reviewed Amazonian *Eugenia*, lack metabolomic or biosynthetic studies [16]. Filling these gaps could reveal genetic resources for a variety of selection and pharmacognosy.

## 5. Chemical Composition

Myrtaceae fruits typically exhibit high water and carbohydrate contents, with lower protein and lipid levels [65]. Nutritionally, they provide diverse organic/inorganic compounds, including minerals, carbohydrates, and lipids [39]. Characteristic aromas from leaves and twigs signal essential oils with potential bioactivities [66], while red–yellow fruit coloration suggests antioxidant properties [6].

Essential oil and leaf/twig extract compositions have been studied for all species: *Eugenia biflora* [67], *E. egensis* [68], *E. flavescens* [69], *E. patrisii* [14], *E. polystachya* [68], *E. protenta* [70], *E. punicifolia* [71] and *E. stipitata* [15]. Fruit and seed chemical profiles are available only for *E. patrisii* [72], and *E. punicifolia* [6,13].

*Eugenia stipitata* fruit pulp and seeds contain diverse minerals: macronutrients (calcium, chlorine, sulfur, phosphorus, magnesium, potassium, sodium) and micronutrients (copper, chromium, iron, manganese, nickel, zinc) [13,73]. Similar profiles occur in *E. patrisii* [72].

Table 3 summarizes element concentrations in ripe fruits and seeds. Data on molecular forms and concentrations are lacking for other Amazonian fruit trees. Compared to *E. patrisii* pulp, *E. stipitata* shows higher mineral values (Table 3).

Protein content in *Eugenia* fruits must account for colorimetric variation during fruit maturation and propagule ripening, which is evolutionarily linked to dispersal [25,74]. Ripening involves cell wall breakdown, energy reserve mobilization, secondary metabolite production (some volatile), and color changes [44,75]. Although this review covers eight fruiting species, protein quantification exists only for *E. stipitata* (5.31% to 11.82% in 100 mg pulp, irrespective of ripe fruit color) and *E. patrisii* (12.7–17.5 g 100 g^−1^) [13,42,64].

Carbohydrate data are available only for *E. stipitata*, *E. patrisii*, and *E. punicifolia*, key to fleshy fruit maturation as primary propagule energy reserves [75]. *E. stipitata* pulp averaged 6.17 ± n.d. across six studies and 18.51 ± 0.20% dw [13]; seeds contained 58.57 ± 2.32% dw [76]. For *E. patrisii*, Aguiar (1996) [72] reported 6.22 mg carbohydrates per 100 mg dry pulp, while Santos et al. (2026) [64] quantified 76.6–79.3 g 100 g^−1^. Seed data are absent, but morphology/anatomy confirms exalbuminous seeds with cotyledonary reserves. Specific saccharides in *E. stipitata* fruits included (mg g^−1^ dw): glucose (7.49 ± 0.26), fructose (17.58 ± 0.80), sucrose (39.01 ± 2.94), maltose (2.03 ± 0.21), 1-kestose (0.27 ± 0.03), and maltotetraose (1.63 ± 0.09) [13]. In *E. punicifolia*, Ramos et al. (2019) [77] identified α-glucose, β-glucose, and sucrose in pulp and seeds.

### 5.1. Specialized Metabolites

Studies on *Eugenia* volatile composition reveal extensive chemical diversity, dominated by terpenes [78]. Alongside structural variety, *Eugenia* compounds are noted for metabolite pharmacological properties [13], including analgesic, anti-inflammatory, antimicrobial, antioxidant, and cytotoxic effects [79].

**Figure 7:**
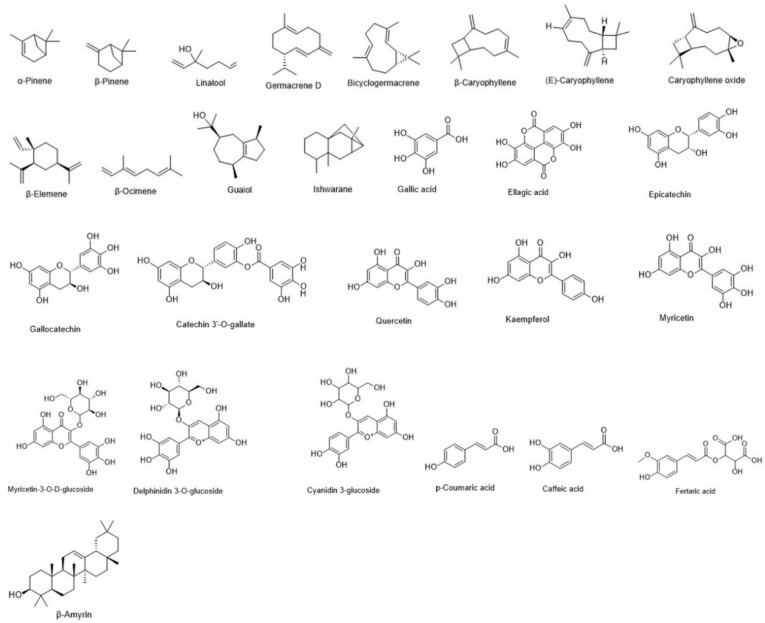
Chemical structure of the major metabolites volatile and non-volatile described for the *Eugenia* Amazonian. Based on Costa et al. 2020 [16].

Key *Eugenia* monoterpenes include α-pinene and β-pinene (Figure 7), prominent in Amazonian *E. biflora* and *E. stipitata*. Pereira et al. (2010) [80] analyzed Myrtaceae leaf essential oils, reporting >27% each for α- and β-pinene in *E. biflora*. In *E. stipitata*, these peaked in fruits: ~17.5% α-pinene in pulp and 15.2% β-pinene in fruits [80]. Linalool (Figure 7) is another abundant monoterpene. Oliveira et al. (2005) [81] first identified it in *E. punicifolia* leaf essential oil, where it comprised 61.2% of the total.

Most volatile compounds in *Eugenia* belong to sesquiterpenes (hydrocarbons and oxygenated derivatives) [68]. Common Amazonian sesquiterpenes include germacrene D (Figure 7), a precursor to various hydrocarbons, detected in six species: *E. patrisii* (15.6%), *E. polystachya* (18.4%) [68], *E. protenta* (15.6%) [70], *E. puncifolia* (5.3%), *E. flavescens* (14.9%) [80], and *E. stiptata* (11.9–38.3%) [15,82]. Its derivative, bicyclogermacrene (Figure 7), predominates *E. flavescens* (11.72%), followed by *E. patrisii* (10%) and *E. punicifolia* (9.8%).

Caryophyllenes, β-caryophyllene, (E)-caryophyllene, caryophyllene oxide (Figure 7), are also widespread. β-Caryophyllene occurs in *E. biflora* [67], *E. egensis* [68], *E. patrisii* [15], *E. puncifolia* [80], and *E. stiptata* [83]. (E)-Caryophyllene appears in leaf essential oils of *E. patrisii* [84], *E. puncifolia* [14], and *E. stiptata* [83]. Other volatiles distributed across Amazonian *Eugenia* include β-elemene (Figure 7): *E. patrisii* (16.9%) [15], *E. protenta* (16.9%) [70], and *E. puncifolia* (25.12%) [14]; while δ-cadinene was found in *E. flavescens* (5.7%) [80], *E. puncifolia* (6%) [71] and *E. stiptata* (12.6%) [82]. β-ocimene (Figure 7) 6.14% [13] and guaiol (Figure 7) 13.77% [17] have been described only in *E. stiptata* species. Ishwarane (Figure 7) is unique to *E. polystachya* among Amazonian species [68].

### 5.2. Non-Volatile Compounds

Gallic acid (Figure 7), essential for tannin synthesis, is prevalent in Amazonian *Eugenia*, extracted from *E. flavescens* leaves [69], *E. punicifolia* [85], *E. protenta* trunks [86], and *E. stipitata* [13]. Other prominent polyphenols include syringic and ellagic acids (Figure 7), common in dicotyledons: syringic acid in *E. punicifolia* leaves/fruits [6,87]; ellagic acid in *E. biflora* leaves [18].

Flavonoid diversity is notable. Catechin and derivatives (e.g., epicatechin, gallocatechin, catechin-3-O-gallate; Figure 7) occur in *E. flavescens* leaves/fruits [69], *E. punicifolia* [18], and *E. stipitata* [13]. Rutin is abundant in *E. flavescens* [69] and *E. punicifolia* [88] leaves. Quercetin (Figure 7) appears in leaves of *E. biflora* [18], *E. flavescens* [69], *E. punicifolia* [85,88], *E. stipitata* [39], and *E. protenta* trunks [86]. Kaempferol (Figure 7) is reported in *E. punicifolia* (leaves/fruits), *E. flavescens* (leaves), and *E. stipitata* (fruits) [6,16]. Myricetin (Figure 7) and derivatives (e.g., myricetin-3-O-β-D-glucoside) occur in *E. stipitata* [39], *E. punicifolia* [18], *E. protenta* [86], and *E. biflora* [18]. Anthocyanins like delphinidin-3-O-glycoside and cyanidin-3-O-glycoside (Figure 7) were characterized in *E. punicifolia* fruits [6].

Organic acids, p-coumaric and caffeic occur in *E. punicifolia* fruits; fertaric acid (Figure 7) in *E. stipitata* fruit extracts [13]. In native *E. protenta*, key terpenes are β-amyrin, sitosterol, and ursolic acid (Figure 7) [86]. *Eugenia*’s rich chemical profiles offer bioindustrial potential.

*Eugenia*’s chemical diversity, from essential oils to phenolics, underscores bioindustrial promise. These data gain significance when linked to ecological roles and genetic variability. Flavonoid/anthocyanin profiles confer nutritional/pharmacological value and may mark stress tolerance. Integrating phytochemicals with morphofunctional/genetic data can guide breeding for resilient, high-value genotypes.

## 6. Biotechnological Potential

Volatile compounds from *Eugenia* fruits, seeds, leaves, and twigs, spanning chemical diversity, have undergone laboratory testing with implications for food, pharmaceutical, cosmetology, and other production lines. *E. stipitata* fruit juice successfully supplemented industrialized apple juice [42]. Its phenolic, flavonoid, and antioxidant richness enhances beverage palatability and nutritional diversity [6].

Sales and Souza (2021) [89] produced Catharina Sour-style craft beer, Souza et al. (2022) [90] wine and Souza et al. (2022) [91] fermented milk drinks at varying concentrations. Neri-Numa et al. (2013) [39] demonstrated pulp compounds’ inhibitory potential against oxidation, mutagenicity, and tumors, it was expanding species utility. *E. stipitata*’s protein/mineral content suggests applications in protein/mineral supplements. *E. punicifolia* fruits lycopene supports candies, effervescent, or supplement tablets.

Predominant classes—phenolics (polyphenols, flavonoids and anthocyanins), organic acids, terpenes—abound across leaves, trunks, fruits (peel, pulp, seeds), yielding bioactive extracts [13,85,87]. Antioxidant properties, linked to phenolics, predominate [92]. In addition, several bioactive organic acids of great biotechnological interest have already been identified in the Amazonian *Eugenia* species. Biotechnologically relevant organic acids like malic and vanillic, acidifiers/flavor/aroma enhancers, occur in Amazonian *Eugenia* [93,94].

*E. punicifolia* pulp exhibits in vitro anti-glycation/antioxidant activity tied to phenolics, plus high ascorbic acid, lycopene, and carotenoids [77]. Despite fruit-tree vocation, leaf/branch volatile/extract bioactivity studies prevail [6]. Most species show potential for drugs, cosmetics, supplements, flavorings, herbicides/fungicides, akin to *Myrciaria dubia*. *E. flavescens* leaf extracts phytotoxically inhibited invasive *Mimosa pudica* and *Senna obtusifolia* germination/hypocotyl–radicle growth (92.1%, 63%; 74–75%, respectively) [69], attributed to gallic acid, quercetin, myricetin—suggesting degradable, soil-safe herbicides.

Silva et al. (2017) [68] confirmed in vitro antioxidant activity in leaf/twig oils of *E. egensis*, *E. flavescens*, *E. patrisii*, and *E. polystachya* cytotoxicity against colon cancer (*E. egensis* excelled in antioxidants; *E. polystachya* in cytotoxicity). *E. stipitata* leaf oil combated Listeria monocytogenes in vitro, rivaling tetracycline [83], indicating natural antibiotic biomolecules. *E. punicifolia* leaf extract allelopathically affected *Lactuca sativa*, *Solanum lycopersicum*, and *Allium cepa* germination time/root growth [95]; it inhibited α-amylase, α-glucosidase, xanthine oxidase (carbohydrate absorption-related) [96], with moderate antitumor activity (melanoma, breast, kidney, lung, ovary, colon, leukemia) [97].

*E. punicifolia* volatiles/extracts show diverse applicability (herbicide, hypoglycemic, antitumor). Further studies could elucidate potential. Like *E. punicifolia*, traditionally hypoglycemic *E. biflora* leaf extract exhibited in vivo antidiabetic effects in rodents via α-amylase/α-glucosidase inhibition and anti-glycation; moderate catechin intake induces hypoglycemia, though excess is cytotoxic [18].

This review synthesizes botanical, resource, and chemical traits of *Eugenia* species, affirming pharmacological, agronomic, and bioindustrial potential. Large-scale cultivation is essential to maximize benefits.

*Eugenia*’s biotechnological uses (food supplements, antimicrobials, herbicides) highlight research translation. Bridging lab data with scalable cultivation, via ecological/genetic integration, will identify optimal genotypes/varieties. This positions *Eugenia* as a model for Amazonian bioeconomy.

## 7. Sustainable Uses and Planting Aspects

Among reviewed species, cultivation data are scarce, as they remain classified as Non-Conventional Food Plants (PANCs). Similarly, information on harvesting, postharvest treatments, storage, transportation, and distribution is limited.

### 7.1. Phenology

Phenological data for reviewed species is scarce; only *Eugenia stipitata* has a complete description [20]. Flowering occurs year-round but peaks during the Amazon dry season (August–December), with perennial fruiting intensified in the Amazonian summer. *E. patrisii* flowers exclusively in the dry period (August–December), yielding contemporaneous fruiting and crop production (Figure 1) [11]. Both species exhibit irregular propagule production among cultivated individuals [11,20]. In *E. stipitata*, flower buds develop into open flowers within 15 days, lasting ~24 h [20]. Flowers emit sweet aromas at opening (attributed to aromatic terpenes), commencing at 4 A.M.—indicative of nocturnal pollination [98].

No floral biology studies exist for Amazonian *E. punicifolia*, but Silva and Pinheiro (2007) [99] described southeastern Brazilian sandbank populations: annual flowering (July–November), fruiting ~1 month later, viability ~24 h, opening ~5:30 A.M. with sweet scent. For *E. biflora*, Amorim and Almeida Jr. (2021) [7] reported to reside in a forest, it was flowering (August–January) and fruiting to February; *E. flavescens* fruits in July; *E. polystachya* flowers in January; *E. protenta* flowers in August, fruits in October.

Southern/southeastern Brazilian *E. egensis* exhibits flowering (June–October) and fruiting (August–December) [37]. Collectively, flowering/fruiting peaks in the latter half of the year, aligning with the Amazon rainy season, supporting annual harvest scheduling.

### 7.2. Planting Systems

Incipient *Eugenia* domestication in the Amazon yields fluctuating fruit quantity, quality, diameter, and palatability [11,20]. Lacking formal protocols, non-native techniques necessitate soil correction, yielding substandard fruit. Cultivated *E. patrisii* averaged 359 ± 33 fruits plant^−1^ (2017–2020) in uncorrected/unfertilized Amazonian red–yellow clay loam [11], rising to 1317 ± 88 with cattle manure fertilization.

Cultivated *E. stipitata* matrices produced up to 400 fruits plant^−1^ in peak seasons and ≥200 in off-peak, oscillations linked to rainfall [20]. Gressler et al. (2006) [25] noted understory Myrtaceae ripen ≤20 fruits simultaneously, constrained by light/nutrients. Ferreira (1992) [43] quantified *E. stipitata* seed number/weight, pericarp diameter/weight over five years; Pacheco et al. (2021) [11] assessed *E. patrisii* height, diameter, dry mass, Dickson quality index, fruit number/mass under cultivation.

Spacing data exist only for *E. patrisii* (1.5 m^2^ plant^−1^; 4 m inter-individual) [11]. Shrubby *E. biflora*, *E. flavescens*, *E. polystachya*, *E. punicifolia*, and *E. stipitata* likely require similar areas. Undomesticated, *E. stipitata*/*E. patrisii* fruit intermittently (September–January), necessitating multiple small harvests [11,20]. *E. patrisii* aerial dry mass increased 409 ± 71 g plant^−1^ (2017–2020) in uncorrected/unfertilized clay loam; data absent for others.

Photosynthetic performance, pigments, relative water content, leaf area/specific leaf area, and limiting nutrients—key for domestication, seedling selection, planting—remain unstudied. Inter-/intraspecific fruit color variation complicates maturity/harvest assessment [25], though, Bohry et al. (2019) [44] validated colorimetric/physicochemical markers for *E. stipitata* genotyping.

Pest data are limited; Souza-Adaime et al. (2017) [74] documented *E. stipitata* orchard infestation by fruit flies (*Bactrocera carambolae*, *Anastrepha obliqua*, *A. fraterculus*, *A. striata* [Tephritidae]; *Neosilba bela*, *N. zadolicha*, *N. glaberrima*, *N. pseudozadolicha* [Lonchaeidae]). Infestations targeted ripe/unripe fruits (ripe preferred), enabling herbivory, oviposition, larval development, and pupation.

Sustainable *Eugenia* use demands integrated approaches blending ecological adaptability, genetic diversity, and phytochemical value. Agroforestry with selected genotypes could bolster climate resilience and community economies. Linking cultivation to genetic/biochemical data transitions experimental systems to scalable solutions for food security, conservation, and innovation.

### 7.3. Soil and Climate Modeling

Despite their heterogeneity, Amazonian soils are predominantly characterized by low concentrations of phosphorus and potassium, as well as calcium and magnesium cations, and high concentrations of silicon, aluminum cations and oxides; in some cases, iron and manganese are also present. These soils are slightly acidic and contain organic carbon [100]. Furthermore, tropical soils in direct contact with large volumes of water collections, featuring alluvial or colluvial sediments, tend to be of recent Quaternary origin and still undergoing weathering [101]. This is coupled with the dependence of one-sixth of Amazonian forest species on flooding regimes for flowering and fruiting [102].

The climate in tropical and subtropical ecosystems, mostly accompanied by temperatures around 25 °C, also drives the selection of species capable of germination, growth, and development in these environments, that is limits to plasticity in photosynthetic generation, gas exchange, and resource uptake, as exemplified by representatives of the Myrtaceae family in the genera *Myrcia* and *Campomanesia* [103].

Under cultivation conditions, *Eugenia patrisii* exhibited a direct relationship between soil organic enrichment and the production of larger and more numerous fruits compared to unfertilized controls, indicating that yields in common Amazonian planting areas would require soil amendments [11]. However, in floodplain areas, soils show higher concentrations of phosphorus and nitrogen [102], while retaining the Amazonian climate in which wild specimens evolved.

The cultivation of herbaceous species and small-stature plants, akin to fruiting *Eugenia* shrubs, has demonstrated efficiency and productivity in flood-prone areas in Brazil [104], North Africa [105], India [106], and Southeast Asia [107]. Thus, *Eugenia* cultivation may be feasible in other tropical and subtropical ecosystems worldwide.

## 8. Conclusions and Future Perspectives

Fruit trees of the genus *Eugenia* are cultivated in Brazil and exhibit morphological, ecological, biochemical, agronomic and genetic traits that favor the domestication and development of high-value bioproducts. These characteristics increase the status of unconventional food plants to potential inputs for diverse economic sectors. To unlock this potential, further research is required to address the gaps in seed biology, germination, ecological strategies, phenology, chemical composition, and biological activity, thereby adding both commercial and scientific value. Expanding in vivo and in vitro germplasm banks, coupled with modern cultivation technologies and seedling production systems, is essential for adapting to and harnessing the genetic diversity of the eight Amazonian species.

The scarcity of information and records on these species hinders advances in new studies and even compromises diversity conservation. Thus, establishing collections emerges as a pathway for maintenance and progress toward Amazonian domestication.

Restoring degraded Amazonian landscapes remains a pressing challenge amidst urban expansion, pastures, and monocultures. Incorporating resilient *Eugenia* species into reforestation and agroforestry systems could strengthen ecological recovery while generating socioeconomic benefits. Ultimately, in the context of biodiversity loss and concentrated food production, these species hold promise not only as future genetic resources, but also as emerging crops with potential acceptance in consumer markets and long-term contributions to food security and sustainability.

## Figures and Tables

**Table 1:** Description of the morphology of *Eugenia* flowers occurring in the Brazilian Amazon.

Species	Inflorescence Description	Reference
*Eugenia biflora*	Inflorescence in racemes, with 1 to 14 axillary flowers on peduncles, isolated or opposite. Bracts and bracteoles lanceolate, sometimes linear, pubescent and persistent. Pedicellate flowers with four rounded or acute sepals, densely pubescent and persistent; four oval petals; hypanthium densely pubescent; staminiferous disk with up to 74 stamens.	[9,36]
*Eugenia egensis*	Inflorescences in axillary racemes, with 1 to 10 single or opposite pedunculate flowers. Persistent bracts and bracteoles. Opposite pedicellate flowers with four deltoid sepals; four oblong, white, glabrous petals, glands present on both surfaces; glabrous hypanthium; staminiferous disk with up to 80 stamens.	[30,37]
*Eugenia flavescens*	Inflorescence in an axillary umbelliform raceme on leaves, with one to seven flowers on a pubescent peduncle, opposite or isolated. Rounded bracts, also pubescent, and ovate to orbicular bracteoles fused only at the base, glabrous and persistent. Pedicellate flowers with four whites, orbicular to obovate petals; four green, ovate sepals; pubescent hypanthium with prominent glands; pubescent staminiferous disk with 61 to 86 stamens.	[9,30]
*Eugenia patrisii*	Inflorescence in axillary or terminal fascicle with one to five flowers on short, lignified peduncles, opposite or isolated. Pink, cylindrical bracts and dark, swollen basal bracteoles. Flowers with long, slender, glabrous pedicels; four green, glabrous sepals, slightly fused at the base; four white, slightly pubescent petals; slightly pubescent hypanthium; staminiferous disk with 68 to 97 stamens.	Authors
*Eugenia polystachya*	Inflorescence in terminal racemes, with 2 to 15 flowers on peduncles, opposite or in triads. Persistent, densely pubescent floral bracts, accompanied by bracteoles. Pedicellate flowers with four densely pubescent and persistent sepals; four white petals also pubescent; hypanthium pubescent; staminiferous disk with up to 88 stamens.	[7,9]

**Table 2:** Description and components of the *Eugenia* fruits occurring in the Brazilian Amazon.

Species	Particularity of Fruit	Reference
*Eugenia biflora*	Globose, black fruit with a hairy surface and inconspicuous glands on the exocarp (Figure 3A). Contains up to five seeds.	[9,36]
*Eugenia egensis*	Globose or elliptical fruit, yellowish or reddish in color, with a glabrous surface and inconspicuous glands on the exocarp. Contains up to four seeds.	[37]
*Eugenia flavescens*	Globose, greenish fruit with a glabrous surface and conspicuous glands on the exocarp. Contains up to three seeds.	[30]
*Eugenia patrisii*	Globose, spherical or pyriform fruit, reddish or purple in color, with a glabrous surface and glands on the exocarp. Contains up to three seeds.	Authors
*Eugenia polystachya*	Globose, brown fruit with a pubescent surface and glands on the exocarp. Contains up to five seeds.	[7,9]
*Eugenia protenta*	Globose fruit, black or yellowish in color, with a glabrous surface and glands on the exocarp. Contains up to three seeds.	[7]
*Eugenia punicifolia*	Ellipsoid or globose fruit, reddish in color, with a glabrous surface and conspicuous glands on the exocarp. Contains up to three seeds.	[30]
*Eugenia stipitata*	Spherical, yellowish fruit with a glabrous surface and glands on the exocarp. Contains up to 20 seeds.	[20]

**Table 3:** List of minerals and their respective concentrations in fruits and seeds of *Eugenia stipitata* and *E. patrisii* (mg·100·g^−1^·dw). According to Leterme et al. (2006) [73], Araújo et al. (2021) [13] and Aguiar (1996) [72].

Mineral	*Eugenia stipitata*		*Eugenia patrisii*	
	Contents (mg·100·g^−1^·dw)			
	Pulp	Seed	Pulp	Seed
Calcium (Ca)	107.16 ± 1.54	22.37 ± 0.29	33.8 ± 0.0	
Cloro (Cl)	−0.1	-	-	
Copper (Cu)	1.12 ± 0.02	0.66 ± 0.03	0.1 ± 0.0	
Chrome (Cr)	0.01 ± 0.0	-	-	
Sulfur (S)	14.0 ± 0.0	-	-	
Iron (Fe)	3.74 ± 0.05	2.29 ± 0.04	0.88 ± 0.0	
Phosphorous (P)	7.0 ± 0.0	-	-	
Magnesium (Mg)	75.65 ± 1.28	35.80 ± 0.60	33.0 ± 0.0	
Manganese (Mn)	0.49 ± 0.02	0.31 ± 0.01	0.11 ± 0.0	
Nickel (Ni)	0.01 ± 0.0	-	-	
Potassium (K)	827.66 ± 14.51	231.99 ± 2.34	275.2 ± 0.0	
Sodium (Na)	118.95 ± 4.43	54.15 ± 0.9	8.2 ± 0.0	
Zinc (Zn)	1.32 ± 0.04	0.74 ± 0.01	0.9 ± 0.0	
Ashes	91.4 ± 1.00%	87.96 ± 0.40%	77 ± 0.0%	

## Data Availability

The original contributions presented in this study are included in the article. Further inquiries can be directed to the corresponding authors.
